# MiR-525-3p Enhances the Migration and Invasion of Liver Cancer Cells by Downregulating ZNF395

**DOI:** 10.1371/journal.pone.0090867

**Published:** 2014-03-05

**Authors:** Fei Pang, Ruopeng Zha, Yingjun Zhao, Qifeng Wang, Di Chen, Zhenfeng Zhang, Taoyang Chen, Ming Yao, Jianren Gu, Xianghuo He

**Affiliations:** 1 Shanghai Medical College, Fudan University, Shanghai, China; 2 State Key Laboratory of Oncogenes and Related Genes, Shanghai Cancer Institute, Renji Hospital, Shanghai Jiao Tong University School of Medicine, Shanghai, China; 3 Shanghai Cancer Hospital, Fudan University, Shanghai, China; 4 Qi Dong Liver Cancer Institute, Qi Dong, China; 5 Department of Experimental Pathology, Shanghai Cancer Institute, Shanghai Jiao Tong University School of Medicine, Shanghai, China; Institute of Molecular and Cell Biology, Biopolis, United States of America

## Abstract

Liver cancer is one of leading causes of cancer-related deaths. A deeper mechanistic understanding of liver cancer could lead to the development of more effective therapeutic strategies. In our previous work, we screened 646 miRNAs and identified 11 that regulate liver cancer cell migration. The current study shows that miR-525-3p is frequently up-regulated in liver cancer tissues, and enhanced expression of miR-525-3p can promote liver cancer cell migration and invasion. Zinc finger protein 395 (ZNF395) is the direct functional target gene for miR-525-3p, and it is frequently down-regulated in liver cancer tissues. High expression of ZNF395 can significantly inhibit while knockdown of ZNF395 expression can markedly enhance the migration and invasion of liver cancer cells, suggesting that ZNF395 suppresses metastasis in liver cancer. Down-regulation of ZNF395 can mediate miR-525-3p induced liver cancer cell migration and invasion. In conclusion, miR-525-3p promotes liver cancer cell migration and invasion by directly targeting ZNF395, and the fact that miR-525-3p and ZNF395 both play important roles in liver cancer progression makes them potential therapeutic targets.

## Introduction

Metastases are the main cause of cancer-related death [1], [2]. Systematically studying the molecular mechanisms of liver cancer metastasis is particularly important for the development of new therapeutic strategies. Tumor metastasis is a multi-stage complex process in which tumor cells move to surrounding or distant tissues after breaking away from the primary tumor. This process involves tumor cell transit through the extracellular matrix (ECM) and the basement membrane of the local blood vessel [3], [4], [5], [6], [7], followed by movement into the host microenvironment [8], [9], [10]. Recent studies have found that in addition to protein coding genes, non-coding RNAs such as miRNAs also play important regulatory roles in the process of metastasis [11], [12], [13], [14], [15], [16]. miRNAs are single-stranded small non-coding RNAs, and their sequences are highly conserved in eukaryote [17]. miRNAs regulate gene expression at the post-transcriptional level by binding to target mRNA [18], [19], [20], and thus participate in various biological process [21], [22]. Meng et al [23] first reported that aberrant expression of miR-21 can mediate liver cancer cell invasion by directly targeting PTEN. Recently, miR-151 and miR-30d are found to be located on genomic fragile sites and are associated with cancer metastasis [24], [25]. Hypoxia-inducible expression of miR-210 regulates VMP1-mediated hypoxia-induced liver cancer cell metastasis [26].

To screen miRNAs involved in liver cancer metastasis, in a previous study, we screened 646 miRNAs using wound healing assay with the live cell imaging system, and 11 miRNAs were found to effectively regulate liver cancer cell migration [27]. In a previous report [28], we identified some copy number variation regions in the genomic DNA of 58 pairs of liver cancer tissues using an SNP Array 6.0. In the present study, we found that miR-525-3p gene is located in a copy number amplified region and it could facilitate liver cancer cell migration in the transwell assay. Additionally, miR-525-3p is frequently up-regulated in liver cancer tissues and regulates liver cancer cell migration and invasion by down-regulating the expression of ZNF395. These findings suggest that miR-525-3p and ZNF395 represent potential targets for liver cancer treatment.

## Materials and Methods

### Human Liver Tumor Samples/Ethics Statement

Human liver cancer and adjacent nontumorous tissues were obtained from the surgical specimen archives of Qidong Liver Cancer Institute, Jiangsu Province, China. All these samples were obtained with written informed consent, and the protocols were approved by the Ethical Review Committee of the WHO Collaborating Center for Research in Human Production authorized by the Shanghai Municipal Government. The specific samples used in this study have been described in previous publication [29].

### Cell Culture

HEK-293T, NCI-H1299, BxPC-3, PANC-1, Hep3B, PLC/PRF/5,HepG2, SK-HEP-1, MCF7, A549, NCI-H460, Tera-1 and Tera-2 were purchased from ATCC; HuH-7 was purchased from Japanese Collection of Research Bioresources (JCRB), SMMC-7721 and BEL-7402 were purchased from Typical culture preservation commission cell bank, Chinese academy of sciences (NCB); MHCC-97L and LM3 were gifts from Zhongshan Hospital, Fudan University (Shanghai, China); SMMC-7721, BEL-7402, MHCC-97L and LM3 used in this study have been described in previous publication [24], [30], [31], [32].

HEK-293T, NCI-H1299, BxPC3, PANC1, Hep3B, PLC/PRF/5, HepG2, HuH-7, HepG2, SK-HEP-1, SMMC-7721, 97L, LM3, BEL-7402 and MCF7 cells were cultured in Dulbecco’s Modified Eagle’s Medium (DMEM) supplemented with 10% fetal bovine serum(FBS). A549 and NCI-H460 cells were cultured in RPMI1640 medium supplemented with 10% FBS. Tera1 and Tera2 cells were cultured in McCoy’s 5a medium supplemented with 15%FBS. All cell lines were cultured in the presence of antibiotics at 37°C with 5%CO_2_.

### RNA Extraction and Quantitative Real-time PCR

Total RNA was extracted using TRIzol reagent (Invitrogen). cDNA was synthesized with the Prime-Script RT reagent Kit (TaKaRa). Real-time PCR was performed with SYBR Premix Ex Taq (TaKaRa). Mature miRNAs were reverse-transcribed and quantified using TaqMan miRNA assays (Applied Biosystems). The probes and primers used for miRNA and mRNA detection are listed in Table S1 and S2.

### Plasmid Vector Constructs

The primary miR-525 sequence was amplified from the genomic DNA of normal tissues and ligated into a PGIPZ vector (Open Biosystem). The ZNF395 ORF sequence was amplified from the ZNF395 vector (FulenGen) and ligated into the pWPXL vector (a gift from Professor Didier Trono). The ZNF395 3′UTR was amplified from the genomic DNA of HK-293T cells and subcloned directly downstream of the stop codon of the luciferase gene in the luciferase reporter vector. Mutant 3′UTR was obtained from the cloned ZNF395 3′UTR using the Quikchange lightning site-directed mutagenesis kit (Agilent). Both the wild-type and mutant 3′UTR sequences were confirmed by sequencing (Invitrogen). The primer sequences are reported in Table S3.

### Lentivirus Production and Cell Transduction

The packaging plasmid psPAX2 and the envelope plasmid pMD2.G were gifts of Professor Didier Trono. Either the pGIPZ-miR-525 or pWPXL-ZNF395 vector was cotransfected with psPAX2 and pMD2.G into HEK293T cells using Lipofectamine 2000 (Invitrogen). Viruses were harvested 48 h after transfection and viral titers were determined. SK-HEP-1 and SMMC-7721 cells were infected with 1×10^6^ recombinant lentivirus transduction units in the presence of 6 µg/mL polybrene (Sigma, MO). SMMC-7721-525, SK-HEP-1-525 and SK-HEP-1-ZNF395 are pools of stable cells, which infected using lentivirus with GFP, stable cell lines used in assays with >95% green fluorescence.

### Oligonucleotide Transfection

Cells were seeded in 6-well plates. After 24 h, 100 pmol of miRNA mimic (Genepharma), inhibitor (Ribobio) or siRNA was transfected using 5 µL lipofectamine RNAiMax reagent (Invitrogen). Cells were harvested 48 h later for transwell assays or luciferase reporter assays. The siRNA sequences are provided in Table S4.

### The Transfection Efficiency Detection

Cells were transfected in 6-well plates using 100 pmol siRNA with FAM (Genepharma) and RNAiMax reagent (Invitrogen), after 20–24 h, transfection efficiency was detected using flow cytometry (FCM).

### Cell Proliferation Assays

Cell proliferation was assessed by the Cell Counting Kit-8 assay kit (CCK-8 Dojindo Corp). 1×10^3^ cells were seeded in each well of 96-well plate and cultured with 90 µL medium, 10 µL CCK-8 was added into each well. After incubated at 37°C for 2 h, the absorbance was detected at 450 nm, and the OD450 value is correlated with the number of live cells.

### Migration and Invasion Assays

Cell migration assay: 5×10^4^ cells were suspended in 200 µl serum-free DMEM medium and seeded into the upper chamber of each insert. Then, 800 µL of DMEM containing 10% FBS was added to a 24-well plate. After incubation at 37°C (SK-HEP-1∶6–8 h; SMMC-7721∶12–16 h), the cells that migrated were fixed and stained for 30 min in a dye solution containing 0.2% crystal violet and 20% methanol. Cell invasion assay: Chambers were uniformly covered with 40–80 µL Matrigel (BD Biosciences) diluted with DMEM to a certain percentage and incubated at 37°C for 2–4 h. Then, 1×10^5^ cells were suspended in 200 µL DMEM and seeded in the upper chambers, and 800 µL DMEM containing 10% FBS was added to the lower chamber. After incubation at 37°C (SK-HEP-1 14–16 h; SMMC-7721 16–18 h), the cells were fixed and stained.

### Luciferase Reporter Assay

HEK293T cells were cultured in a 96-well plate and transfected with 50 ng pluc-3′UTR, 10 ng Renilla luciferase plasmid and 5 pmol miR-525-3p mimic or negative control. After 48 h incubation, luciferase activity was detected using the dual-luciferase reporter assay system (Promega).

### Western Blot Assay

Cell lysates were separated by 10% SDS-PAGE, transferred to a nitrocellulose membrane (Bio-Rad), blocked with phosphate-buffered saline containing Tween-20 and 5% nonfat milk for 1 h, and incubated with ZNF395 polyclonal antibody (Santa Cruz) (1∶1000) or β-actin (Sigma) (1∶10000). The antibody complex was detected using enhanced chemiluminescence (Pierce).

### Statistical Analysis

All results are presented as the mean ± standard error of the mean (SEM). Differences between groups were analyzed using Student’s t test (two-tailed, p<0.05 was considered statistically significant).

## Results

### High Expression of miR-525-3p Enhances the Migration and Invasion of Liver Cancer Cells

**Figure 1:**
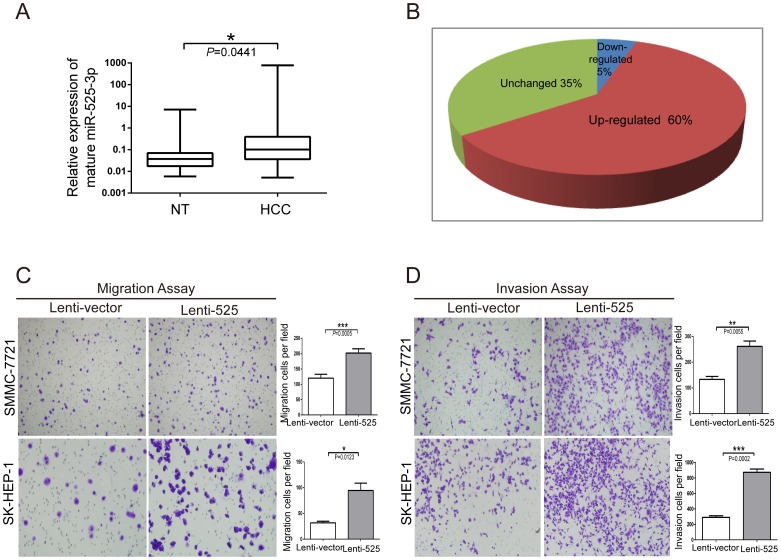
miR-525-3p is frequently up-regulated in liver cancer, and high expression of miR-525 promotes SMMC-7721 and SK-HEP-1 migration and invasion. (**A**) miR-525-3p expression was determined by TaqMan Real time PCR in liver cancer and adjacent noncancerous liver tissue samples (NT) (n = 136). The miR-525-3p expression level was normalized to U6b snRNA. The relative expression of miR-525-3p is reported in a box plot with a log_10_ y-axis scale. The ends of the boxes define the 25^th^ and 75^th^ percentiles, and the line indicates the median. Statistical analysis was performed by a paired t-test, and the stars indicate statistically significant results at P<0.05(*). (**B**) The pie chart shows the proportions of liver cancer tissues in which miR-525-3p expression was up-regulated (60%), down-regulated (5%) and unchanged (35%). (**C**) Transwell migration assays of SMMC-7721 and SK-HEP-1 cells expressing miR-525 or vector control. (**D**) Transwell invasion assays of SMMC-7721 and SK-HEP-1 cells expressing miR-525 or vector control. Representative images are shown on the left, and 3 randomly selected fields are quantified on the right. The values shown here represent the mean ± S.E.M, and the stars indicate statistically significant results at P<0.01, ** or P<0.001, ***.

To explore the role of miR-525-3p in liver cancer development, the expression level of miR-525-3p was detected in 136 liver cancer and paired adjacent noncancerous liver tissues (NT). miR-525-3p was significantly up-regulated in liver cancer tissues (Figure 1A), with up-regulation observed in 60% of liver cancer tissues (Figure 1B). In addition, we examined the expression of miR-525-3p in various cancer cell lines. The results showed that the expression level of miR-525-3p is relatively high in liver cancer cell lines (Figure S1 in File S1). To examine the biological function of miR-525 in HCC, stable cell lines expressing miR-525 was constructed and named SMMC-7721-525 and SK-HEP-1-525 (Figure S2A in File S1). CCK-8 assays suggested that miR-525 did not influence HCC cell growth (Figure S3 in File S1), while transwell assays indicated that over-expression of miR-525 promoted SK-HEP-1 and SMMC-7721 migration and invasion (Figure 1C, D).

### MiR-525-3p Post-transcriptionally Down-regulates ZNF395 Expression by Directly Targeting its 3′UTR

**Figure 2:**
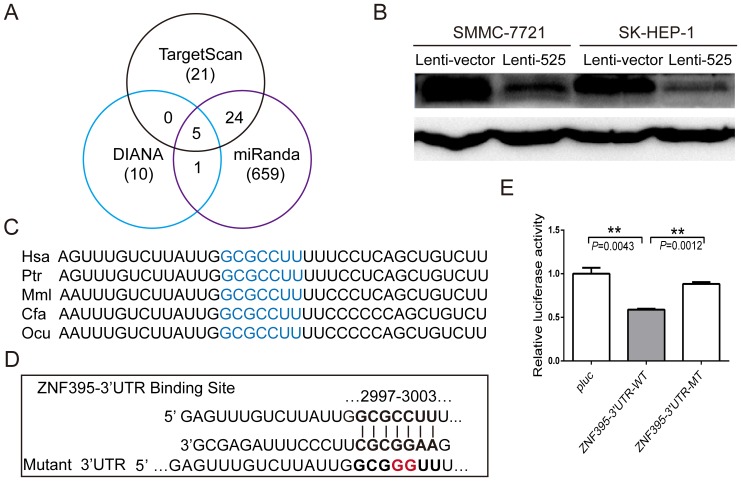
miR-525-3p post-transcriptionally down-regulates ZNF395 expression by directly targeting its 3′UTR. (**A**) Schema of the candidate genes produced by different prediction algorithms. Each circle represents one prediction algorithm and the number of genes that it predicted, and the numbers listed in the regions where the circles overlap represent the number of genes predicted by both algorithms. (**B**) Western blot assays of the ZNF395 protein levels in SMMC-7721 and SK-HEP-1 cells infected with miR525 or vector control. (**C**) Potential binding sequences for miR525-3p within the ZNF395 3′UTR of human (Hsa), chimpanzee (Ptr), rhesus (Mml), dog (Cfa) and rabbit (Ocu). Seed sequences are underlined. (**D**) Luciferase reporter plasmids were constructed by inserting the ZNF395 3′UTR and corresponding fragments into the region downstream of the luciferase gene. The predicted binding sequences for miR-525-3p are shown, including the wild-type full length UTR (WT) or mutant (MT, underlined) binding sites. (**E**) Relative luciferase activity was assayed after HEK-293T cells were co-transfected with WT or MT ZNF395 3′UTR reporter plasmids and miR-525-3p. The values are reported as the mean ± S.E.M, and the stars indicate statistical significance at P<0.01, **.

We next sought determine the target genes of miR-525-3p. Potential target genes were predicted using the miRNA prediction algorithms TargetScan, Miranda and DIANA. Joint predictions of each two software were combined and 30 candidate genes were found (Figure 2A). The expression levels of these genes were detected after miR-525-3p mimics were introduced into SK-HEP-1 and SMMC-7721. RANBP10, NACC1, IRF1, ZNF395 and MLST8 were inhibited by more than 40% (Figure S4 in File S1). To understand whether these five genes are related to liver cancer, the expression levels of these genes were detected in 12 pairs of liver cancer tissue samples. RANBP10, IRF1 and ZNF395 were found to be down-regulated in liver cancer tissues, while the expression levels of NACC1 and MLST8 remained unchanged (Figure S5 in File S1).

To understand whether RANBP10, IRF1 and ZNF395 are potential functional target genes of miR-525-3p, these genes were knockdown using siRNA. The results showed that interference with IRF1 expression could inhibit the migration of SMMC-7721 and SK-HEP-1 cells, a phenotype that is inconsistent with the phenotype of miR-525-3p over-expression. Knockdown of endogenous RANBP10 and ZNF395 expression was able to promote the migration of SMMC-7721 and SK-HEP-1 cells. This phenotype is consistent with the phenotype of miR-525-3p over-expression. (Figure S6 in File S1). Then, the RANBP10 and ZNF395 protein levels were assayed in SK-HEP-1-525 and SMMC-7721-525 cells to determine whether miR-525 over-expression could inhibit the expression of these proteins. The results showed that the ZNF395 protein level was significantly decreased in SMMC-7721-525 and SK-HEP-1-525 cells (Figure 2B). Whereas the RANBP10 protein level was not significantly affected (Figure S7 in File S1). Thus, ZNF395 was selected for further study.

The ZNF395 3′UTR contains a binding site for miR-525-3p (predicted by TargetScan), and the binding sequence is highly conserved in various species such as human, chimpanzee, rhesus monkey, dog and rabbit (Figure 2C). To determine whether miR-525-3p directly regulates ZNF395 by targeting its 3′UTR, a ZNF395 full-length 3′UTR luciferase reporter vector was constructed. Luciferase activity decreased significantly after cotransfection with the miR-525-3p mimic and luciferase 3′UTR constructs. In contrast, the relative luciferase activity did not change significantly when mutant binding sites were introduced (Figure 2D, E), suggesting that miR-525-3p could regulate ZNF395 expression by directly binding to its 3′UTR.

### ZNF395 is Frequently Down-regulated in Liver Cancer and can Inhibit Liver Cancer Cell Migration and Invasion

**Figure 3:**
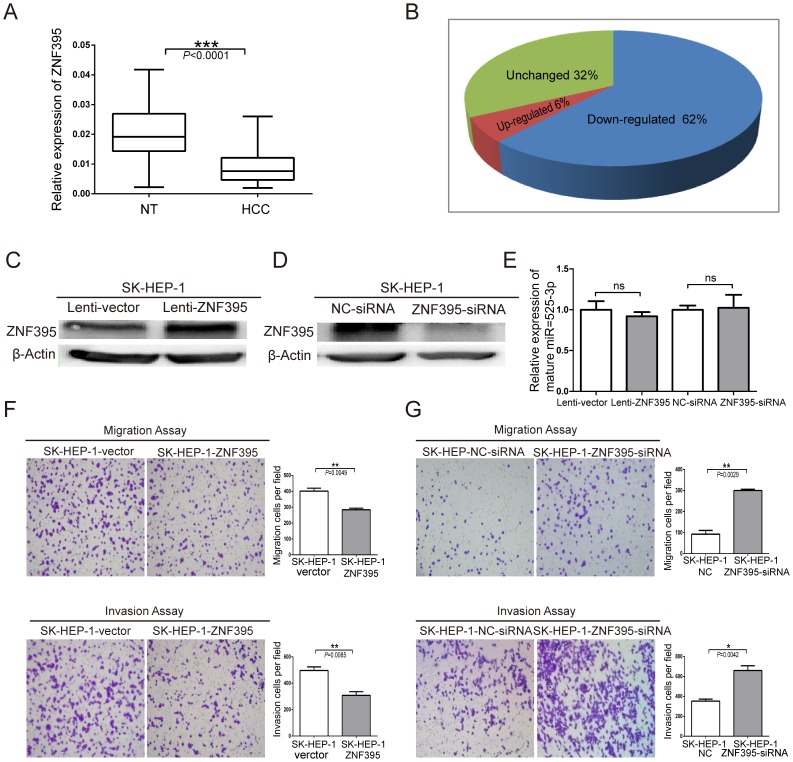
ZNF395 is frequently down-regulated in liver cancer and can inhibit SK-HEP-1 cell migration and invasion. (**A**) Relative expression levels of ZNF395 in HCC tissues or matched noncancerous tissues (NT) were determined using quantitative reverse-transcription PCR assays. (**B**) The pie chart shows the proportions of liver cancer samples in which ZNF395 was down-regulated (62%), unchanged (32%), and up-regulated (6%). ZNF395 expression was determined by TaqMan Real time PCR in liver cancer and adjacent noncancerous liver tissues (NT) (n = 136), and ZNF395 expression levels were normalized to Gapdh. The relative expression of ZNF395 is presented in a box plot. Statistical analysis was performed with a paired t-test. (**C, D**) Lentivirus-mediated over-expression or siRNA-induced knockdown of ZNF395 expression was detected by Western blot in SK-HEP-1. ((**E**) Relative expression levels of mature miR-525-3p in ZNF395 over-expression or knockdown SK-HEP-1 cells. (**F**) Transwell migration and invasion assays of SK-HEP-1cells stably expressing ZNF395 or vector control. (**G**) Transwell migration and invasion assays of SK-HEP-1 cells after the knockdown of endogenous ZNF395. Representative images are shown on the left, and 3 randomly selected fields are quantified on the right. Error bars represent the S.E.M; stars indicate statistical significance at P<0.05, *; P<0.01, **; or P<0.001, ***.

Zinc finger protein 395 is a member of the Krüppel C2H2-type zinc finger protein family, and most proteins in this family function as transcriptional activators or inhibitors. ZNF395 has not been previously reported to be involved in liver cancer or tumor metastasis. Therefore, the effects of ZNF395 on the development and progression of liver cancer and the mechanism underlying those effects should be investigated. ZNF395 was significantly down-regulated in liver cancer tissues (Figure 3A), with down-regulation observed in 62% of liver cancer tissues (Figure 3B). To clarify the effect of ZNF395 in liver cancer cell migration and invasion, the stable cell line SK-HEP-1-ZNF395 was established (Figure S2B in File S1) and siRNAs against ZNF395 were ordered, the knockdown efficiency of ZNF395-siRNAs was determined by real -time PCR (Figure S2C in File S1), and the transfection efficiency of SK-HEP-1 and SMMC-7721 were 89.5% and 97.0% (Figure S2 D, E, F, G in File S1).

The ZNF395 expression level was detected by western blot assay (Figure 3C, D). And relative expression levels of mature miR-525-3p was not obviously changed in ZNF395 over-expression or knockdown SK-HEP-1 cells (Figure 3E), so that can rule out the possibility of a cross-regulation of miR-525 by ZNF395. This assay showed that over-expression of ZNF395 could significantly inhibit SK-HEP-1 migration and invasion (Figure 3F), while interference with endogenous ZNF395 expression could significantly promote the migration and invasion of SK-HEP-1 cells (Figure 3G).

### Restoration of ZNF395 Inhibits miR-525–induced Liver Cancer Cell Migration and Invasion

**Figure 4:**
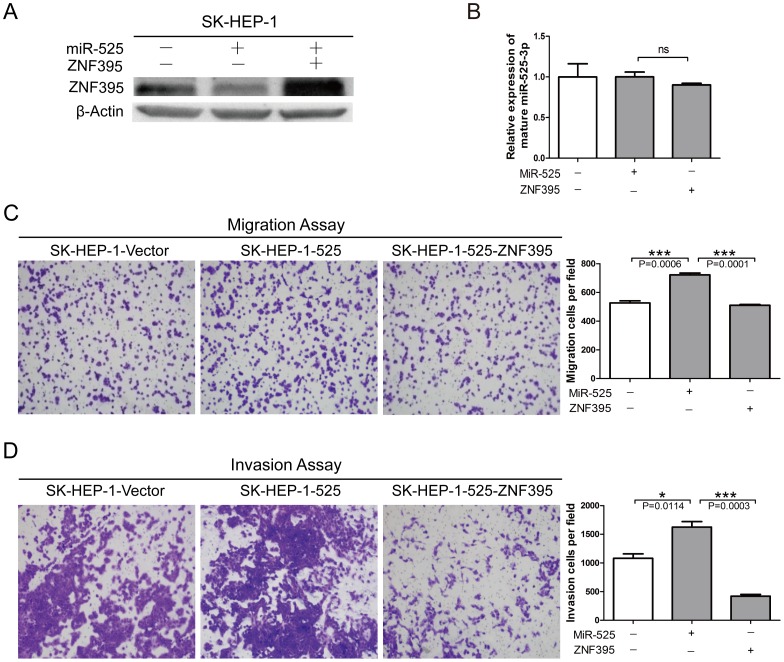
Restoration of ZNF395 expression inhibits miR-525–mediated liver cancer cell migration and invasion. (**A**)Western blot assays for SK-HEP-1-vector or SK-HEP-1-525 cells with or without the reintroduction of ZNF395. (**B**) Relative expression levels of mature miR-525-3p in ZNF395 over-expression or knockdown SK-HEP-1 cells. (**C, D**) Transwell migration, invasion assays for SK-HEP-1-vector or SK-HEP-1-525 cells with or without the reintroduction of ZNF395. These results are representative of at least three independent experiments. Statistical analysis was performed with the student’s t-test. Error bars represent S.E.M, and stars indicate statistical significance at P<0.05, *; and P<0.001, ***.

In an attempt to test whether ZNF395 is the direct functional mediator of miR-525 induced migration and invasion, a ZNF395 lentiviral vector containing the full-length ORF but excluding the 3′UTR was introduced into the SK-Hep1-525 stable cell line (Figure 4A). Relative expression levels of mature miR-525-3p were not obviously changed in SK-HEP-1-525 and SK-HEP-1-525-ZNF395 cells (Figure 4B). The results showed that high ZNF395 expression was able to offset miR-525-induced changes in SK-HEP-1 migration and invasion (Figure 4C, D). These findings indicated that ZNF395 is a direct functional target gene for miR-525-3p.

## Discussion

miR-525 is located on chromosome 19q13.42 without a host gene. The expression level of miR-525-3p increased 6.8-8-fold in two cisplatin-resistant germ cell tumor cell lines [33]. Wang et al [34] detected miRNA expression using miRNA microarray and found that miR-525-3p was up-regulated in three pairs of liver cancer tissues. There are no reports on the function and mechanism of miR-525-3p in cancer. In this study, for the first time, we found that elevated expression of miR-525-3p promotes liver cancer cell migration and invasion. ZNF395 is identified as a direct functional target gene for miR-525-3p.

ZNF395 is a member of the Krüppel C2H2 type zinc finger protein family. It is widely expressed and was originally identified as a transcription factor regulating human papilloma virus (HPV) expression; thus, it is also known as HPV-binding factor (PBF) and can bind to the HPV promoter region [35]. ZNF395 is expressed in hypoxia-induced glioblastoma cell lines and in the blood vessels of adult glioblastoma tissues [36]. Tsukahara et al [37] found that ZNF395 is a tumor-associated antigen. ZNF395 was also shown to regulate the PI3K/Akt pathway to inhibit cell growth [38] by activating caspase-3 to promote apoptosis [39]. There are no reports of ZNF395 being involved in tumor metastasis.

**Figure 5:**
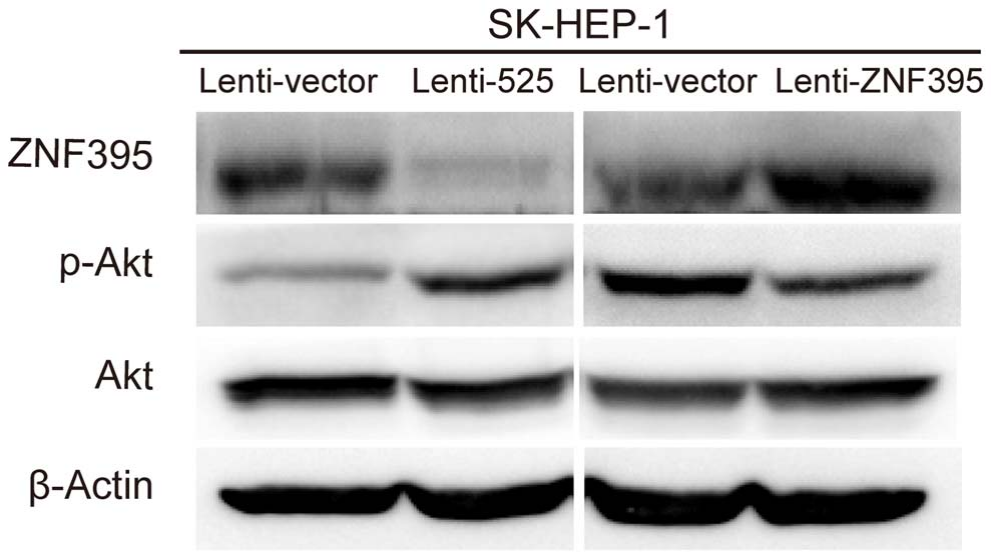
Over-expression of ZNF395 and miR-525 can regulate PI3-K/Akt pathway through alteration of the status of p-Akt. Western blot analysis of PI3-K/Akt pathway in SK-HEP-1-525 and SK-HEP-1-ZNF395 cells.

We report for the first time that ZNF395 is frequently down-regulated in liver cancer tissues and that miR-525-3p can specifically target the ZNF395 3′UTR. Over-expression of ZNF395 can inhibit liver cancer cell migration and invasion, while restoration of ZNF395 inhibits miR-525–mediated liver cancer cell migration and invasion. Over-expression of ZNF395 and miR-525 have no effect in NF-κB, MAPK pathway, but can regulate PI3-K/Akt pathway through alteration of the status of p-Akt (Figure 5, Figure S8 in File S1).

In summary, this study shows that high expression of miR-525-3p promotes liver cancer cell migration and invasion. ZNF395 is a direct functional target gene of miR-525-3p; miR-525-3p promotes liver cancer cell migration and invasion by down-regulating the expression of ZNF395. This finding reveals a new mechanism of liver cancer metastasis and new targets for the treatment of liver cancer.

## Supporting Information

Table S1 **The sequences of miRNA probes.**
(DOCX)

Table S2 **The real-time PCR primers for predicted potential target gene candidates.**
(DOCX)

Table S3 **Primers for miR-525, ZNF395, ZNF395 3′UTR and mutant 3′UTR cloning.**
(DOCX)

Table S4 **SiRNA sequences against IRF1, RANBP10 and ZNF395.**
(DOCX)

File S1 **Supporting information on results obtained, containing Figure S1, S2, S3, S4, S5, S6, S7 and S8.** Figure S1. The expression levels of miR-525-3p in various cancer cell lines. miR-525-3p expression were determined by TaqMan Real time PCR in cancer cell lines. The expression levels were normalized to U6 snRNA. Figure S2. The expression levels of stable cell lines highly expressing miR-525 and ZNF395. (A) Relative expression levels of mature miR-525 in SMMC-7721-525 and SK-HEP-1-525, ABI TaqMan miRNA assays were used and data was normalized by U6b snRNA. (B) The relative expression level of ZNF395 in SK-HEP-1 or vector control, data was normalized by gapdh. (C) Knockdown efficiency of ZNF395-siRNAs. The mixture of ZNF393-siRNA-1, ZNF393-siRNA-2, and ZNF393-siRNA-3 was named ZNF393-siRNA-1+2+3. (D, E, F, G) Transfection efficiency of FAM-siRNA in SK-HEP-1 and SMMC-7721 cells. Stars are indicated to show significance, P<0.01, **; P<0.001, ***. Figure S3. miR-525 has no significant effects on HCC cell growth. Cell proliferation assays for SMMC-7721 (A) and SK-HEP-1 (B) cells infected with lentivirus expressing miR525 or vector control. Cell counting kit-8 (CCK-8) assay was used to assess Cell proliferation. The mean of values are plotted as shown, and the bars indicate S.E.M in triplicate. P = ns (not significant) by Student’s *t*-test. Figure S4. The expression levels of predicted potential target gene candidates. Data was normalized by gapdh, and presented as mean±S.E.M. Figure S5. The expression levels of candidate genes in liver cancer tissues. Expression of (A) RANBP10, (B) IRF1, (C) ZNF395, (D) NACC1 and (E) MLST8 were determined by quantitative real-time PCR assays in liver cancer and adjacent noncancerous liver tissues(NT) (n = 12). Data was normalized by gapdh, stars are indicated to show significance, P<0.05, *; P<0.001, ***. Figure S6. Interference IRF1, RANBP10 and ZNF395 expression can inhibit or promote SMMC-7721 and SK-HEP-1 cells migration. (A) Interference IRF1 expression could inhibit SMMC-7721 and SK-HEP-1 cells migration. Interference RANBP10 (B) and ZNF395 (C) expression could promote SMMC-7721 and SK-HEP-1 cells migration. Figure S7. RANBP10 expression is not obviously changed in miR-525 over-expressed cells. Western blot assays of the RANBP10 expression in SK-HEP-1 and SMMC-7721 cells infected with miR525 or vector control. Figure S8. Over-expression of MiR-525 and ZNF395 have no effect in NF-κB, MAPK pathway or EMT markers. Western blot analysis of NF-κB (A), MAPK (B) pathway and EMT markers (C) in SK-HEP-1-525 and SK-HEP-1-ZNF395 cells.
(DOC)
