# Supplementary material for: MiR-525-3p Enhances the Migration and Invasion of Liver Cancer Cells by Downregulating ZNF395
Source: PLoS One. 2014 Mar 5;9(3):e90867. doi: 10.1371/journal.pone.0090867 (PMC3944804; doi:10.1371/journal.pone.0090867)
Supplement: Table S1 — The sequences of miRNA probes. (DOCX) [file pone.0090867.s001.docx]

**Supplementary Table**

**Table S1 The sequence of miRNA probes**

| **Assay Name** | **AB Assay ID** | **Target Sequence** |
| --- | --- | --- |
| hsa-miR-525-3p | 002385 | GAAGGCGCUUCCCUUUAGAGCG |
| U6b SnRNA | 001093 | CGCAAGGAUGACACGCAAAUUCGUGAAGCGUUCCAUAUUUUU |
