# Supplementary material for: MiR-525-3p Enhances the Migration and Invasion of Liver Cancer Cells by Downregulating ZNF395
Source: PLoS One. 2014 Mar 5;9(3):e90867. doi: 10.1371/journal.pone.0090867 (PMC3944804; doi:10.1371/journal.pone.0090867)
Supplement: Table S2 — The real-time PCR primers for predicted potential target gene candidates. (DOCX) [file pone.0090867.s002.docx]

**Supplementary Table**

**Table S2 The real-time PCR primers for predicted potential target gene candidates**

| **Name** | **Sense Sequence (5’ to 3’)** | **Antisense Sequence** **(5’ to 3’)** |
| --- | --- | --- |
| CDH11-F | GTTCTTGGCCCCAAGTTACA | TGTGGATTTCTGCTGCAAAG |
| ZNF238-F | TCTGAGCGAGCAGAGACAC | GGTCCTTGTAAAAGAGGTGGAAA |
| PRPF4B-F | AGGAAAGGTCCAGTCTGGTATG | TGTCTAGTCCGTTCTTTGGATCT |
| SDCBP-F | CTGCTCCTATCCCTCACGATG | GGCCACATTTGCACGTATTTCT |
| PDCD10-F | GCCCCTCTATGCAGTCATGTA | AGCCTTGATGAAAGCGGCTC |
| KCNA5-F | GTAACGTCAAGGCCAAGAGC | GGGAGGAAAGGAGTGAAAGG |
| CSDE1-F | AAACCAGAATGACCCATTGCC | ATTTGTCACGTCGGTCTGTTG |
| GPR88-F | ACTTGCTCCTGGCACTGATCCC | TCCTGACACTGTCCTGCCTTGC |
| INHBB-F | TGCGGGTCAAAGTGTACTTCC | ACTGCACGTCTAGGTTGAGTC |
| RBM14-F | AGGCAGTCAGAGAACCCAGGAC | AGGCTAGCAGGACGACGATGAG |
| PPP1R16B-F | ACTCACAGCCCTACACCAGTGC | GTCCACAGCTCGTTGTCCTTGG |
| NACC1-F | CTCTCCCGGCTGAACTTATCA | AGCGTGTTCCGGTCAAAGAA |
| KPNB1-F | CCACTTTCCTTGTGGAACTGT | CTCTGCTGATATTGTGCCTTGA |
| MTUS2-F | TGACCACGACCACAAAGTCCAAG | CCACACAGTTGCCATCACAATCC |
| EBF1-F | TGGGGTTCGTGGAGAAGGAA | CACGTAGAAATCCTGCTCCG |
| ASCC2-F | AGCAGCAATTCCCGAAATGGA | AGGAACTCTTCGATGAAGCCC |
| PLEKHO2-F | CCAGGGAACGGCTATATCGG | CCCAGCACCTTGTTCAACAG |
| THTPA-F | ATGGCCCAGGGCTTGATTG | ATCCACTATCCTCTCGTCGTC |
| IRF1-F | ATGCCCATCACTCGGATGC | CCCTGCTTTGTATCGGCCTG |
| FAM160B2-F | CACATGCCTGCTGAGACCG | CCACCCGACTCTTCTTGCTC |
| ANK3-F | TCCAGTGGGTCTGAGCAAAAGC | GACCGTTCGCTGTTACGAGTGG |
| C1orf144-F | ACCCTTCCAGTCAAGTCCCTAGC | AGATCCTGGTTGGCCTGTCG |
| HES1-F | TCAACACGACACCGGATAAAC | GCCGCGAGCTATCTTTCTTCA |
| ZNF-395-F | TCATGGCTTTGAGACCGATCC | CCACAATGGAGCGCAGAACT |
| MLST8-F | GGGACTTGAAAACAGACCACA | CCGTCAGATTCCAGACATAGCA |
| RANBP10-F | GGCTCAAGGCGTCAACATGA | GGAGCAGAACGAATGCCCAT |
| TLX3-F | ATCTGCGAGCTGGAAA | GATGGAGTCGTTGAGGC |
| TOLLIP-F | TGGGCCGACTGAACATCAC | GTGGATGACCTTATTCCAGCG |
| TEC-F | TTGGAGGAGAAGGTTCATCGG | TCCCTTTCACACTAACTGGGT |
| IER2-F | ACTGGTCCCGAGCAAGAAAG | CGACTTCGGATGACGCTCC |
| IGF2BP1-F | GCGGCCAGTTCTTGGTCAA | TTGGGCACCGAATGTTCAATC |
