# Supplementary material for: MiR-525-3p Enhances the Migration and Invasion of Liver Cancer Cells by Downregulating ZNF395
Source: PLoS One. 2014 Mar 5;9(3):e90867. doi: 10.1371/journal.pone.0090867 (PMC3944804; doi:10.1371/journal.pone.0090867)
Supplement: Table S3 — Primers for miR-525, ZNF395, ZNF395 3′UTR and mutant 3′UTR cloning. (DOCX) [file pone.0090867.s003.docx]

**Supplementary Table**

**Table S3 Primers for miR-525, ZNF395, ZNF395 3’UTR and mutant ZNF395 3’UTR cloning**

| **Name** |  | **Sequence(5’ to 3’)** |
| --- | --- | --- |
| MiR-525-F | Lateral | TTTTCTTTCTGGAGGCGAAA |
| MiR-525-R |  | ATTTTGAGCCAAGCGTTCAC |
| MiR-525-F | Inside | CCGCTCGAGTGGAGGCGAAACTCAGGA |
| MiR-525-R |  | GCGACGCGTCCATCATCCAAGTCTTAA |
| ZNF395-3’UTR-WT-F | Lateral | CTCTGCTTGTTCTCCCAGCC |
| ZNF3953’UTR-WT-R |  | CATTGGGAAGCGTTACCTACTG |
| ZNF395-3’UTR-WT-F | Inside | CCCATCGATGGGAAGAAGGCCTGCCAGCG |
| ZNF395-3’UTR-WT-R |  | CGCGGATCCGCGGGCAAAGGACAGGCACAC |
| ZNF395-3’UTR-MT-F |  | GTTTGTCTTATTGGCGGGTTTTTCCTCAGCTGTC |
| ZNF395-3’UTR -MT-R |  | GACAGCTGAGGAAAAACCCGCCAATAAGACAAAC |
| ZNFORF-F |  | CGCGGATCCGCGATGGCGAGTGTCCTGTC |
| ZNFORF-R |  | CCGGAATTCCGGTAGTCCAGAAAGCGCTG |
