# Supplementary material for: MiR-525-3p Enhances the Migration and Invasion of Liver Cancer Cells by Downregulating ZNF395
Source: PLoS One. 2014 Mar 5;9(3):e90867. doi: 10.1371/journal.pone.0090867 (PMC3944804; doi:10.1371/journal.pone.0090867)
Supplement: Table S4 — SiRNA sequences against IRF1, RANBP10 and ZNF395. (DOCX) [file pone.0090867.s004.docx]

**Supplementary Table**

**Table S4 siRNA sequences against IRF1, RANBP10 and ZNF395**

| siRNA | Sequence (5' to 3') |
| --- | --- |
| siRNA-Negative control  siRNA-IRF1-1  siRNA-IRF1-2  siRNA-IRF1-3 | UUCUCCGAACGUGUCACGUTT  CCAGAUCCCAUGGAAGCAUTT  CCCUGAUACCUUCUCUGAUTT  GCACCAGUGAUCUGUACAATT |
| siRNA-RANBP10-1  siRNA-RANBP10-2 | GUGGCAUUUAUUACUUUGATT  CGUGCAUCAUGGGUAUUGUTT |
| siRNA-RANBP10-3 | GCAAACAGAACCACAGUAATT |
| siRNA-ZNF395-1 | CUGGGCAGAAGGUUUAUGUTT |
| siRNA-ZNF395-2 | GGAACAUCGAUGUCCCAAATT |
| siRNA-ZNF395-3 | GCACAUUCAGGCAGAUCAUTT |
