# Supplementary figures and images for: MiR-525-3p Enhances the Migration and Invasion of Liver Cancer Cells by Downregulating ZNF395
Source: PLoS One. 2014 Mar 5;9(3):e90867. doi: 10.1371/journal.pone.0090867 (PMC3944804; doi:10.1371/journal.pone.0090867)

**Supplementary Figures**

**Figure S1**


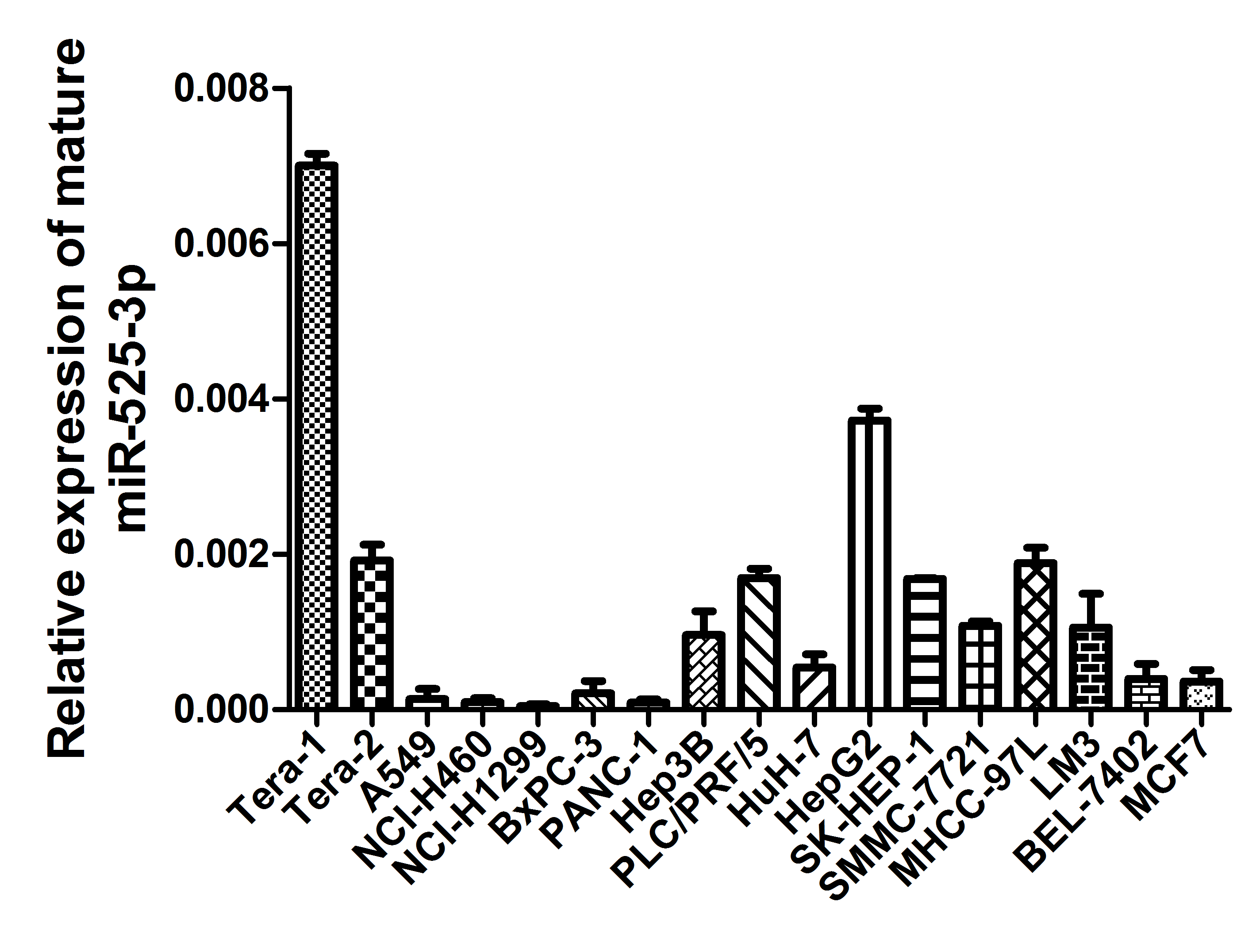


**Figure S2**

**
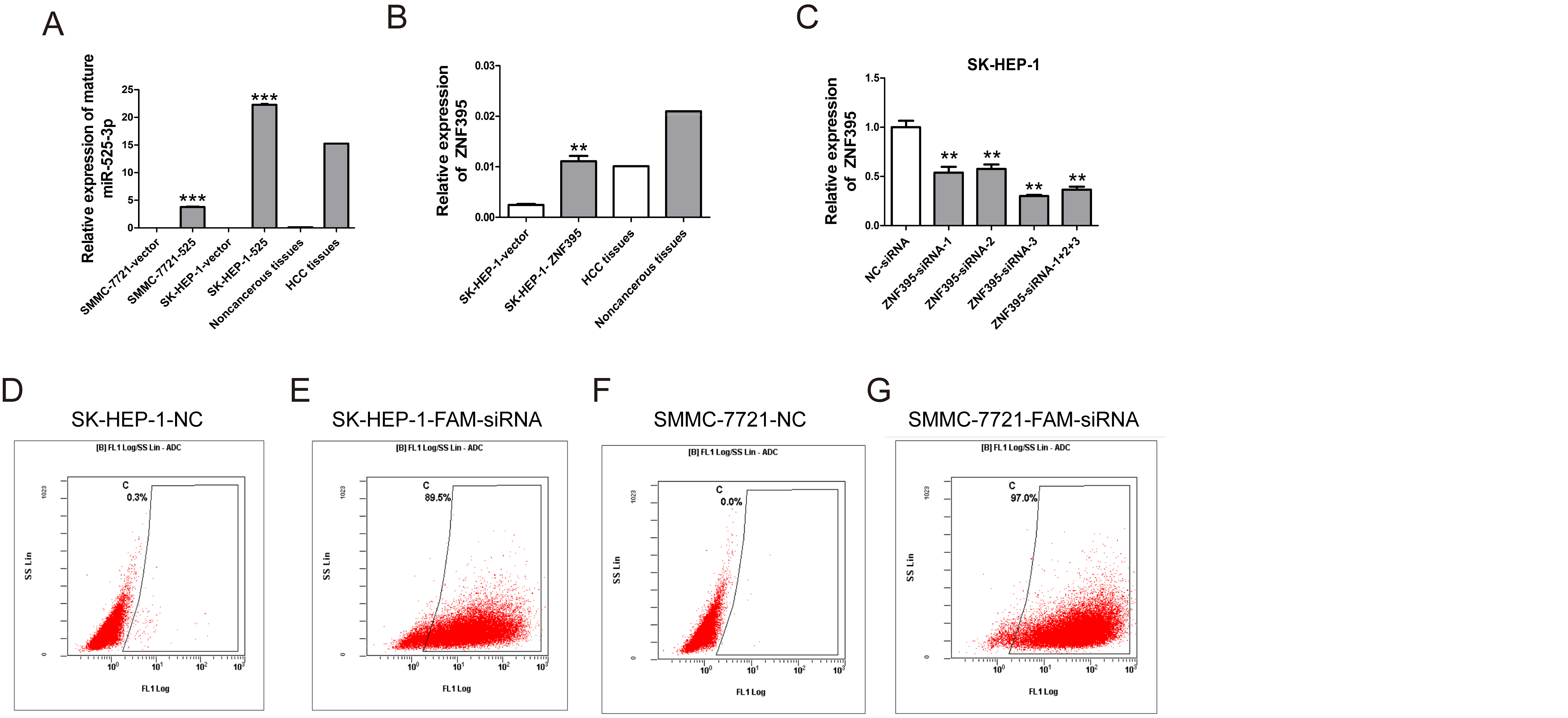
**

**Figure S3**

**
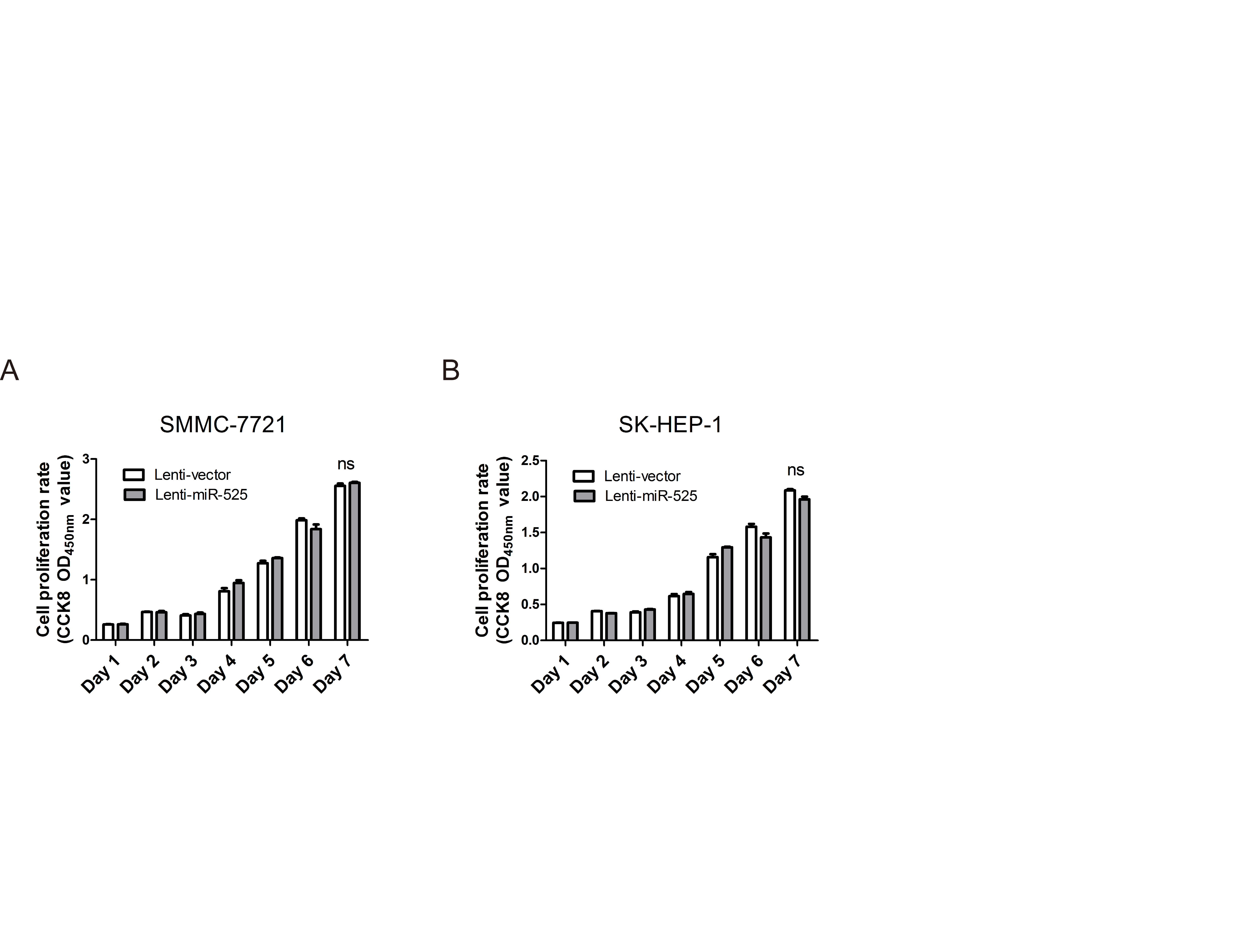
**

**Figure S4**


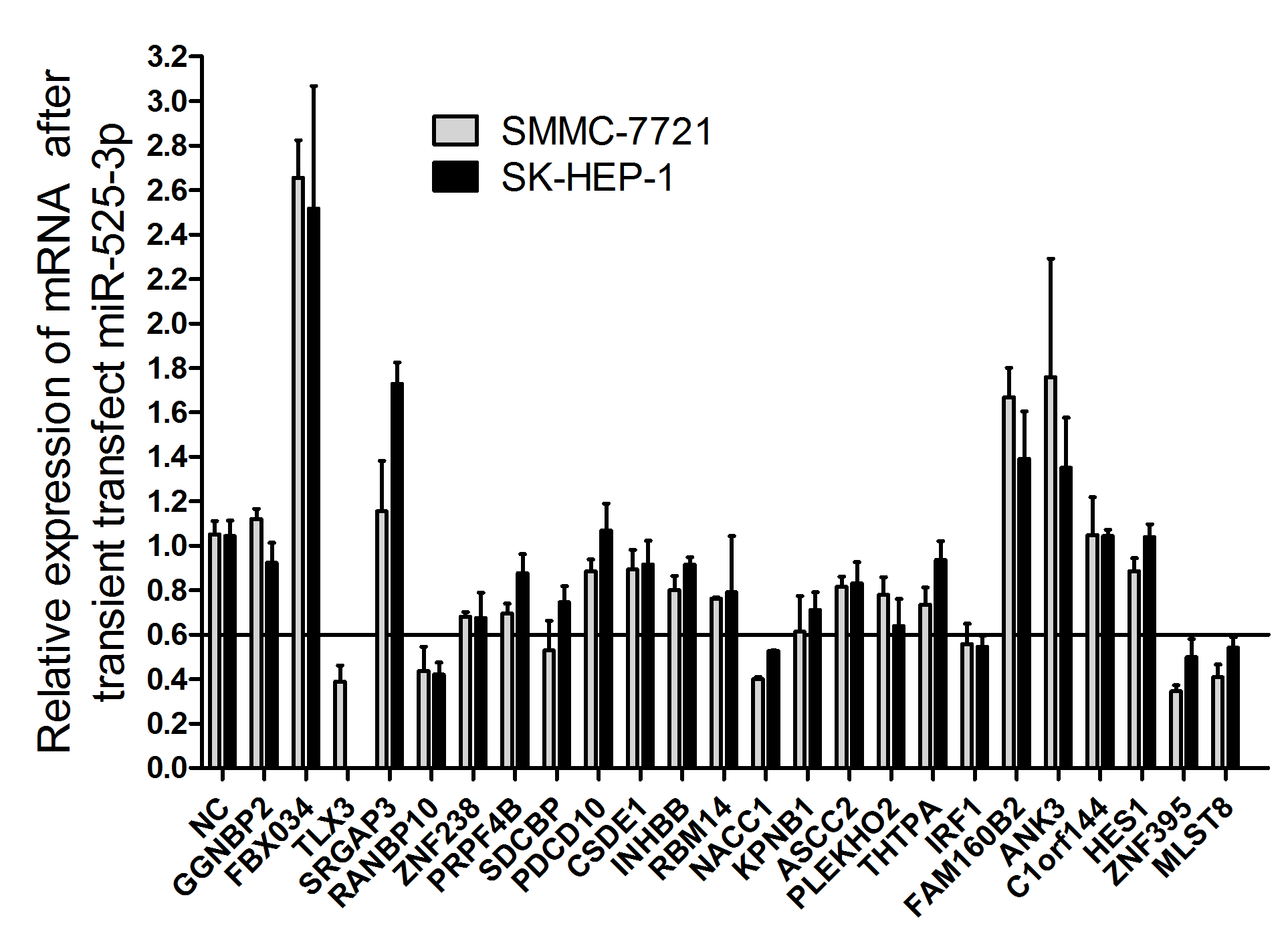


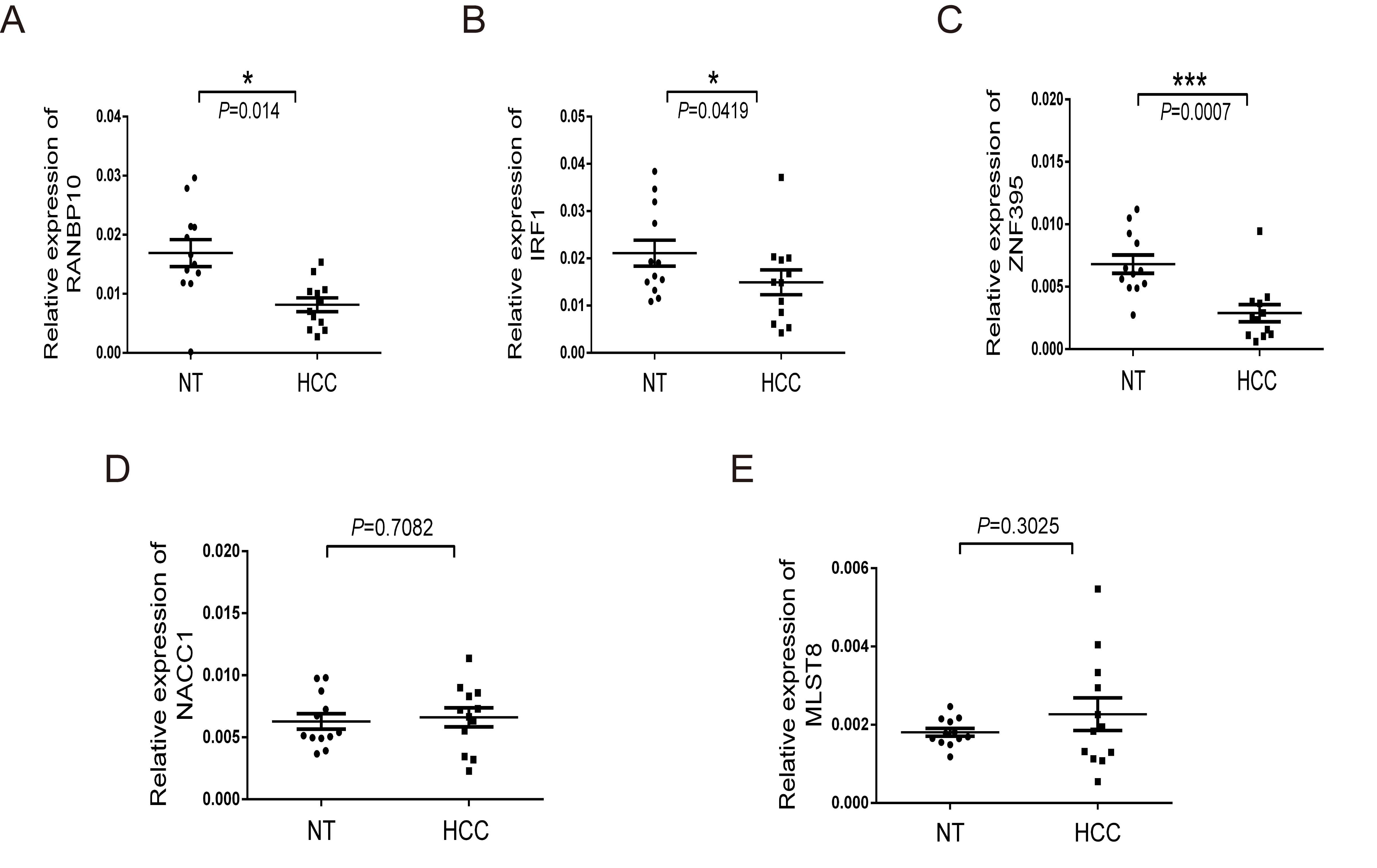
**Figure S5**


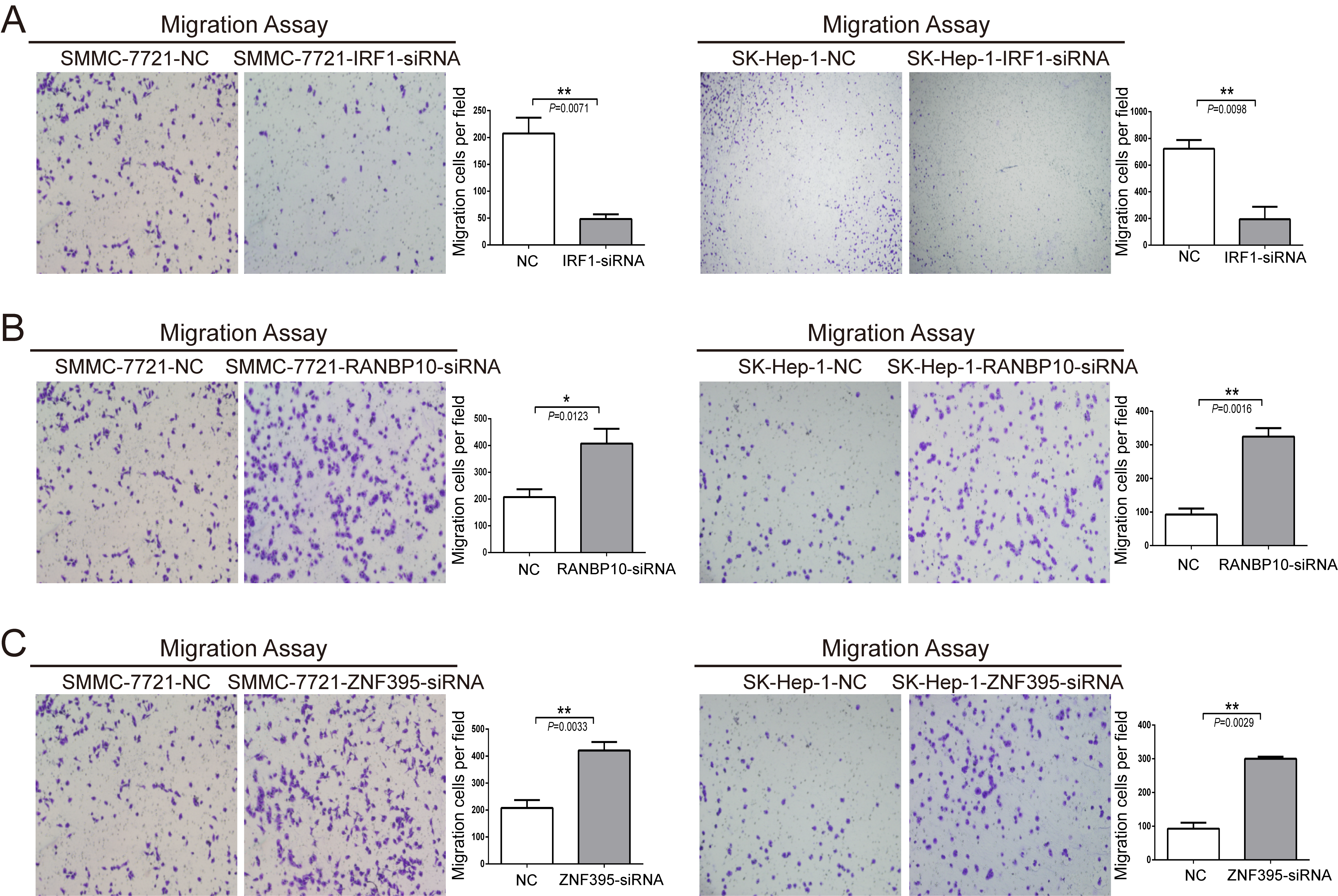
**Figure S6**

**Figure S7**


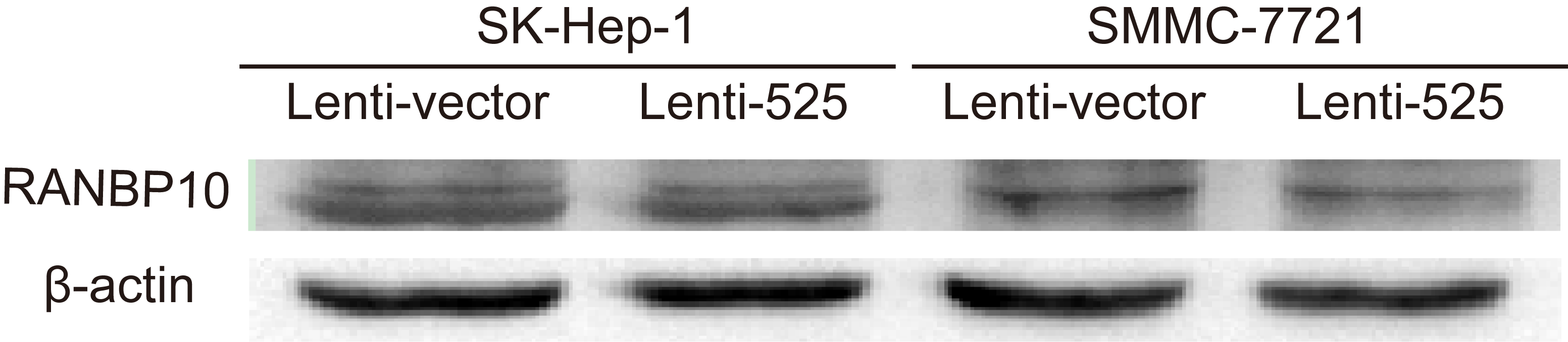


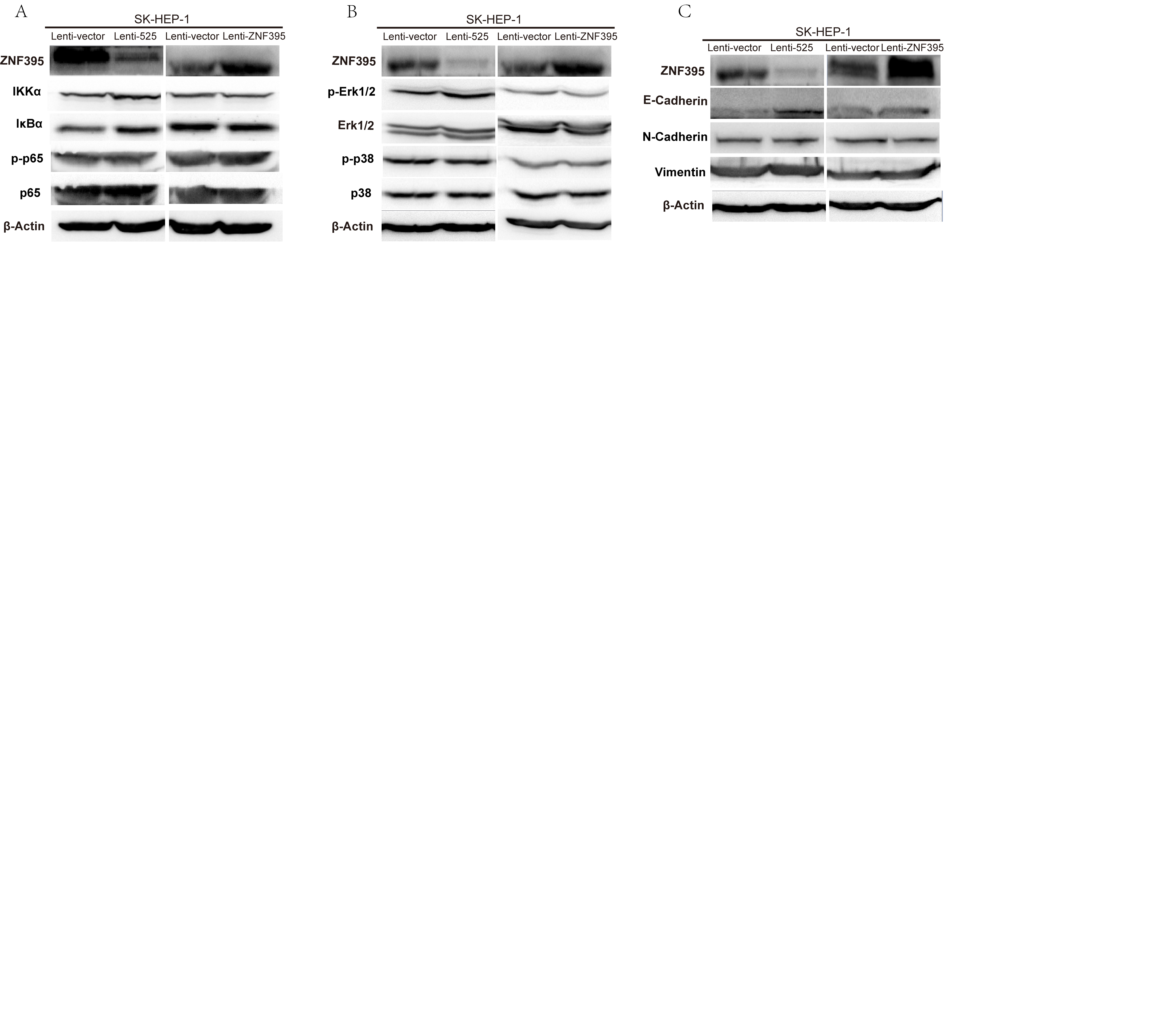
**Figure S8**

Supplement: File S1 — Supporting information on results obtained, containing Figure S1, S2, S3, S4, S5, S6, S7 and S8. Figure S1. The expression levels of miR-525-3p in various cancer cell lines. miR-525-3p expression were determined by TaqMan Real time PCR in cancer cell lines. The expression levels were normalized to U6 snRNA. Figure S2. The expression levels of stable cell lines highly expressing miR-525 and ZNF395. (A) Relative expression levels of mature miR-525 in SMMC-7721-525 and SK-HEP-1-525, ABI TaqMan miRNA assays were used and data was normalized by U6b snRNA. (B) The relative expression level of ZNF395 in SK-HEP-1 or vector control, data was normalized by gapdh. (C) Knockdown efficiency of ZNF395-siRNAs. The mixture of ZNF393-siRNA-1, ZNF393-siRNA-2, and ZNF393-siRNA-3 was named ZNF393-siRNA-1+2+3. (D, E, F, G) Transfection efficiency of FAM-siRNA in SK-HEP-1 and SMMC-7721 cells. Stars are indicated to show significance, P<0.01, **; P<0.001, ***. Figure S3. miR-525 has no significant effects on HCC cell growth. Cell proliferation assays for SMMC-7721 (A) and SK-HEP-1 (B) cells infected with lentivirus expressing miR525 or vector control. Cell counting kit-8 (CCK-8) assay was used to assess Cell proliferation. The mean of values are plotted as shown, and the bars indicate S.E.M in triplicate. P = ns (not significant) by Student’s t-test. Figure S4. The expression levels of predicted potential target gene candidates. Data was normalized by gapdh, and presented as mean±S.E.M. Figure S5. The expression levels of candidate genes in liver cancer tissues. Expression of (A) RANBP10, (B) IRF1, (C) ZNF395, (D) NACC1 and (E) MLST8 were determined by quantitative real-time PCR assays in liver cancer and adjacent noncancerous liver tissues(NT) (n = 12). Data was normalized by gapdh, stars are indicated to show significance, P<0.05, *; P<0.001, ***. Figure S6. Interference IRF1, RANBP10 and ZNF395 expression can inhibit or promote SMMC-7721 and SK-HEP-1 cells migration. (A) Interference I [file pone.0090867.s005.doc]
